# Symptom clusters and quality of life among patients with thyroid cancer during early survivorship: a national multicenter cross-sectional study

**DOI:** 10.1093/oncolo/oyag313

**Published:** 2026-09-24

**Authors:** Jie Gao, Xin Wang, Jiayan Cao, Yanyan Chen, Yi Tu, Xiaohua Song, Yingchun Zhou, Duo Tao, Lianjie Zhang, Jing Zhao

**Affiliations:** Tianjin Medical University Cancer Institute & Hospital, National Clinical Research Center for Cancer, Tianjin’s Clinical Research Center for Cancer, Tianjin Key Laboratory of Cancer Prevention and Therapy, Tianjin 300060, China; Tianjin Medical University Cancer Institute & Hospital, National Clinical Research Center for Cancer, Tianjin’s Clinical Research Center for Cancer, Tianjin Key Laboratory of Cancer Prevention and Therapy, Tianjin 300060, China; Tianjin Medical University Cancer Institute & Hospital, National Clinical Research Center for Cancer, Tianjin’s Clinical Research Center for Cancer, Tianjin Key Laboratory of Cancer Prevention and Therapy, Tianjin 300060, China; The Third Clinical Medical College of Xinjiang Medical University, Urumqi, Xinjiang Uygur Autonomous Region 830011, China; Zhejiang Cancer Hospital, Hangzhou Institute of Medicine (HIM), Chinese Academy of Sciences, Hangzhou, Zhejiang 310022, China; Hunan Cancer Hospital/The Affiliated Cancer Hospital of Xiangya School of Medicine, Central South University, Changsha, Hunan 410000, China; Chongqing University Cancer Hospital, Chongqing Cancer Research Institute, Chongqing 400030, China; Sun Yat-sen Memorial Hospital, Sun Yat-sen University, Guangzhou, Guangdong 510120, China; The First Bethune Hospital of Jilin University, Changchun, Jilin 130031, China; Tianjin Medical University Cancer Institute & Hospital, National Clinical Research Center for Cancer, Tianjin’s Clinical Research Center for Cancer, Tianjin Key Laboratory of Cancer Prevention and Therapy, Tianjin 300060, China

**Keywords:** symptom clusters, quality of life, thyroid cancer, follow-up period

## Abstract

**Background:**

Patients with thyroid cancer commonly experience multiple symptoms during early survivorship after thyroidectomy. However, evidence regarding symptom clusters during this period remains limited, hindering the development of targeted interventions to support recovery and improve quality of life.

**Objective:**

This study aimed to explore symptom clusters among patients with thyroid cancer at 1-month postoperative follow-up and analyze their associations with quality of life.

**Design:**

A national multicenter cross-sectional study.

**Setting and participants:**

A total of 1093 patients with thyroid cancer from 29 hospitals in 24 provinces, municipalities, and autonomous regions in China were enrolled for this study. Participants completed the questionnaire between June and December 2024.

**Methods:**

Symptom burden was assessed using the M.D. Anderson Symptom Inventory–Thyroid Cancer module, and quality of life was measured using the European Organization for Research and Treatment of Cancer Quality of Life Questionnaire. Exploratory factor analysis was used to identify symptom clusters. Mann–Whitney *U* tests and Kruskal–Wallis *H* tests were performed to examine differences in quality of life across demographic and clinical characteristics. Multiple linear regression analysis was conducted to identify factors associated with quality of life.

**Results:**

Three symptom clusters were identified during early survivorship following thyroidectomy: a psychological-somatic symptom cluster, a thyroid hormone-related symptom cluster, and a surgery-related symptom cluster. In the multiple linear regression model, all 3 symptom clusters were independently associated with poorer quality of life, with the psychological-somatic symptom cluster showing the strongest association (β = −0.596, 95% CI, −0.713, −0.581). Female sex (β = −0.051, 95% CI, −2.795, −0.253) and total thyroidectomy (β = −0.058, 95% CI, −2.746, −0.390) were also independently associated with poorer quality of life.

**Conclusions:**

Symptom clusters are prevalent among patients with thyroid cancer during early survivorship and are significantly associated with poorer quality of life. The psychological-somatic symptom cluster showed the strongest association, while female patients and those undergoing total thyroidectomy may be at increased risk for poorer quality of life. Early identification of symptom clusters and vulnerable patient groups may facilitate more targeted postoperative assessment and supportive management.

Implications for Practice Identification of symptom clusters during early survivorship supports a shift from individual symptom management toward a symptom cluster–based approach. Particular attention should be given to the psychological-somatic symptom cluster, which showed the strongest association with quality of life. Routine follow-up should include screening for emotional distress, fatigue, sleep disturbance, pain, and appetite-related symptoms. Risk-stratified and multidisciplinary survivorship care, especially for female patients and those undergoing total thyroidectomy, may facilitate targeted support, optimize resource allocation, and improve recovery outcomes.

## Introduction

With advances in medical diagnostic technologies, the incidence of thyroid cancer has increased steadily worldwide.^1^ In 2022, approximately 821,173 new cases were diagnosed globally, accounting for 4.1% of all newly diagnosed cancers.^2^ China alone contributed more than half of the global thyroid cancer burden, with approximately 466,100 new cases reported.^3^ Although thyroid cancer is associated with a favorable prognosis, with a 5-year survival rate of up to 92.9% in China,^4^ the growing population of survivors has made survivorship care an increasingly important clinical issue.

Nevertheless, many patients with thyroid cancer experience persistent physical and psychological symptoms during the early postoperative recovery period, which may substantially impair quality of life. The first month following thyroidectomy represents a clinically sensitive transition phase characterized not only by surgical recovery but also by endocrine adaptation and psychosocial adjustment.^5^^,^^6^ During this period, fluctuations in thyroid hormone levels, physiological stress responses, and changes in daily functioning may contribute to complex and interrelated symptom experiences. Fatigue^7^^,^^8^ and sleep disturbance^9–11^ are among the most frequently reported symptoms during early survivorship. In addition, patients may experience surgery-related and disease-specific symptoms, including voice changes, scar discomfort, autonomic nervous system disturbances, and psychological distress.^12^

Emerging evidence suggests that these symptoms rarely occur independently but may co-occur as symptom clusters, which are increasingly recognized as multidimensional phenomena shaped by shared biological, psychological, and treatment-related processes.^13^ Compared with individual symptoms, symptom clusters may exert a greater impact on patients’ functioning and quality of life because symptoms within a cluster can interact and mutually reinforce one another. Consequently, symptom cluster research has become an important approach for understanding the complexity of symptom experiences and developing more targeted symptom management strategies.

Previous studies have identified several symptom clusters among patients with thyroid cancer. For example, Li et al.^14^ identified four symptom clusters in patients with thyroid cancer during the first 1-3 days after surgery, including throat–voice, numbness–emotional, swallowing-related, and somatic stress symptom clusters. In contrast, de Rooij et al.^15^ reported that patients with thyroid cancer during long-term survivorship exhibited psychological, fatigue–pain–sleep disturbance, and gastrointestinal symptom clusters. These findings suggest that the composition and structure of symptom clusters may vary according to treatment exposure, survivorship stage, and study characteristics such as sample size and patient population. However, current evidence regarding symptom clusters during the early survivorship phase of thyroid cancer remains limited, particularly during the first postoperative month, a critical period marked by ongoing physical recovery and endocrine adaptation. Notably, many patients with thyroid cancer report inadequate symptom support and feelings of neglect during follow-up care,^16^^,^^17^ highlighting the clinical importance of improving the understanding of symptom experiences during recovery. Furthermore, most existing studies have been conducted using relatively small or single-center samples, which may limit the stability and generalizability of the identified symptom cluster structures.

To address these gaps, this national multicenter cross-sectional study aimed to identify prevalent symptom clusters among patients with thyroid cancer at the 1-month postoperative follow-up and to examine factors associated with quality of life. The findings may provide clinically relevant evidence for risk stratification, targeted symptom management, and the optimization of survivorship care following thyroidectomy.

## Methods

### Study design

This study was a national, multicenter, cross-sectional study in China and approved by the Ethics Committee of Tianjin Medical University Cancer Institute and Hospital (Approval number: bc20240073, Approval date: June 6, 2024). Patients with thyroid cancer were recruited consecutively via convenience sampling from 29 hospitals across 24 provinces, municipalities, and autonomous regions in China between June and December 2024.

### Participants and settings

This study adhered to the principles outlined in the Declaration of Helsinki. All eligible participants in this study were recruited during their first postoperative month follow-up visit in the outpatient setting, and written informed consent was obtained. Inclusion criteria were as follows: diagnosis of thyroid cancer confirmed by histopathological examination and consistent with the Guidelines for the Diagnosis and Treatment of Thyroid Carcinoma^18^; performed surgical operation for thyroid cancer for the first time; no obstacle in language comprehension, communication, or literacy ability; age ≥ 18 years; voluntary participation in the study. Exclusion criteria were as follows: severe mental illness; combined with other malignant tumors and serious diseases. Withdrawal criteria: voluntary withdrawal from the study for various reasons.

### Instruments

Sociodemographic variables included age, gender, marital status, residence, literacy level, occupation, income level, type of medical insurance, and so on. Disease-related variables included clinical stage, pathological type, presence of metastasis, extent of thyroidectomy, thyrotropin value, and so on.

#### M.D. Anderson symptom inventory-thyroid cancer module (MDASI-THY)

Gning et al.^19^ developed and initially validated MDASI-THY specifically for use in patients with thyroid cancer in 2009. In 2022, Chinese scholars Hu et al.^20^ translated into the Chinese version of MDASI-THY with 25 items. In addition to the basic 13 cancer symptom items, a specific thyroid cancer symptom-specific module was added, including 6 symptoms, which are hoarseness, feeling hot, feeling cold, palpitations, dysphagia, diarrhea or constipation. Satisfactory reliability was obtained in the Chinese population validation with Cronbach’s alpha coefficients of 0.827-0.954.

#### European organization for research and treatment of cancer quality of life questionnaire (EORTC QLQ-C30)

The scale was developed by the European Organization for Research and Treatment of Cancer in 1993.^21^ The Chinese version of the scale was translated by Wan et al. in 2005.^22^ The scale has 30 items, including five functional domains of physical functioning, role functioning, emotional functioning, cognitive functioning and social functioning, one domain of global health status, 3 symptom domains of fatigue, nausea and vomiting, and pain, and six single items of shortness of breath, insomnia, loss of appetite, constipation, diarrhea, and financial difficulties. The QLQ-C30 summary score was used as an indicator of overall quality of life and was derived from the mean scores of all functional and symptom subscales, excluding the global health status and financial difficulty domains.^23^ The summary score ranges from 0 to 100, with higher scores indicating better quality of life. The Chinese version has good reliability and validity.^22^

### Sample size

The sample size was determined based on the methodological requirements for exploratory factor analysis, which generally recommend a sample size of 5-10 times the number of items included in the analysis. Given the 69 items assessed in this study, and allowing for a 20% potential attrition rate, the minimum required sample size was estimated to be 863 participants to ensure the robustness of the findings.

### Data collection

Between June and December 2024, on-site data collection was conducted. Each hospital appointed a dedicated research coordinator tasked with participant recruitment and questionnaire administration. All team members at each site completed a unified training and certification covering study objectives, standardized workflows, data management, and quality assurance procedures. Participants completed a structured paper-based questionnaire at their first routine postoperative follow-up visit. Designated coordinators immediately reviewed questionnaires to identify and resolve omissions by consulting participants on-site. Questionnaires that remained incomplete after on-site verification were excluded from the final analysis. Consequently, only participants with complete questionnaire data were included, and no imputation for missing data was performed.

### Statistical analysis

IBM SPSS version 23.0 software was used for statistical analyses. Categorical variables such as sociodemographic and clinical variables were expressed as frequency and percentage. Normally distributed numerical variables were expressed as mean and standard deviation, whereas non-normally distributed variables were presented as median and interquartile range. Symptoms with a prevalence of less than 30% were excluded prior to exploratory factor analysis (EFA) because low-prevalence symptoms may provide insufficient variance and reduce the stability and interpretability of the factor structure. This approach is consistent with previous oncology symptom cluster study.^24^ EFA using principal component analysis with varimax rotation was conducted to identify symptom clusters and obtain a simpler and more interpretable factor structure with clearer factor separation. Sampling adequacy and suitability for factor analysis were evaluated using the Kaiser–Meyer–Olkin (KMO) test and Bartlett’s test of sphericity. A KMO value >0.50 and a significant Bartlett’s test (*P *< .05) were considered indicative of acceptable factorability. Factors were retained based on eigenvalues ≥1, scree plot examination, and clinical interpretability. Items with factor loadings < 0.50 or substantial cross-loadings were sequentially removed, and EFA was repeated iteratively until a stable factor structure was achieved. Each factor was required to contain at least 2 symptoms. Cronbach’s α coefficients were calculated to evaluate the internal consistency reliability of the identified symptom clusters. The Mann–Whitney *U* test and Kruskal-Wallis H test were used to compare quality of life across different demographic and clinical characteristics. Variables showing statistical significance in univariate analyses were subsequently entered into multiple linear regression models to identify independent predictors of quality of life. Statistical significance was defined as a 2-tailed *P *< .05.

## Results

### Regional distribution of participating hospitals and enrolled patients

This study involved 29 hospitals from 24 provinces, municipalities, and autonomous regions across China. The participating hospitals were distributed across seven geographic regions: 7 in North China, 2 in Northeast China, 6 in East China, 3 in Central China, 3 in South China, 3 in Northwest China, and 5 in Southwest China. Detailed distribution of participating hospitals and patient enrollment is shown in Figure 1.

**Figure 1.**
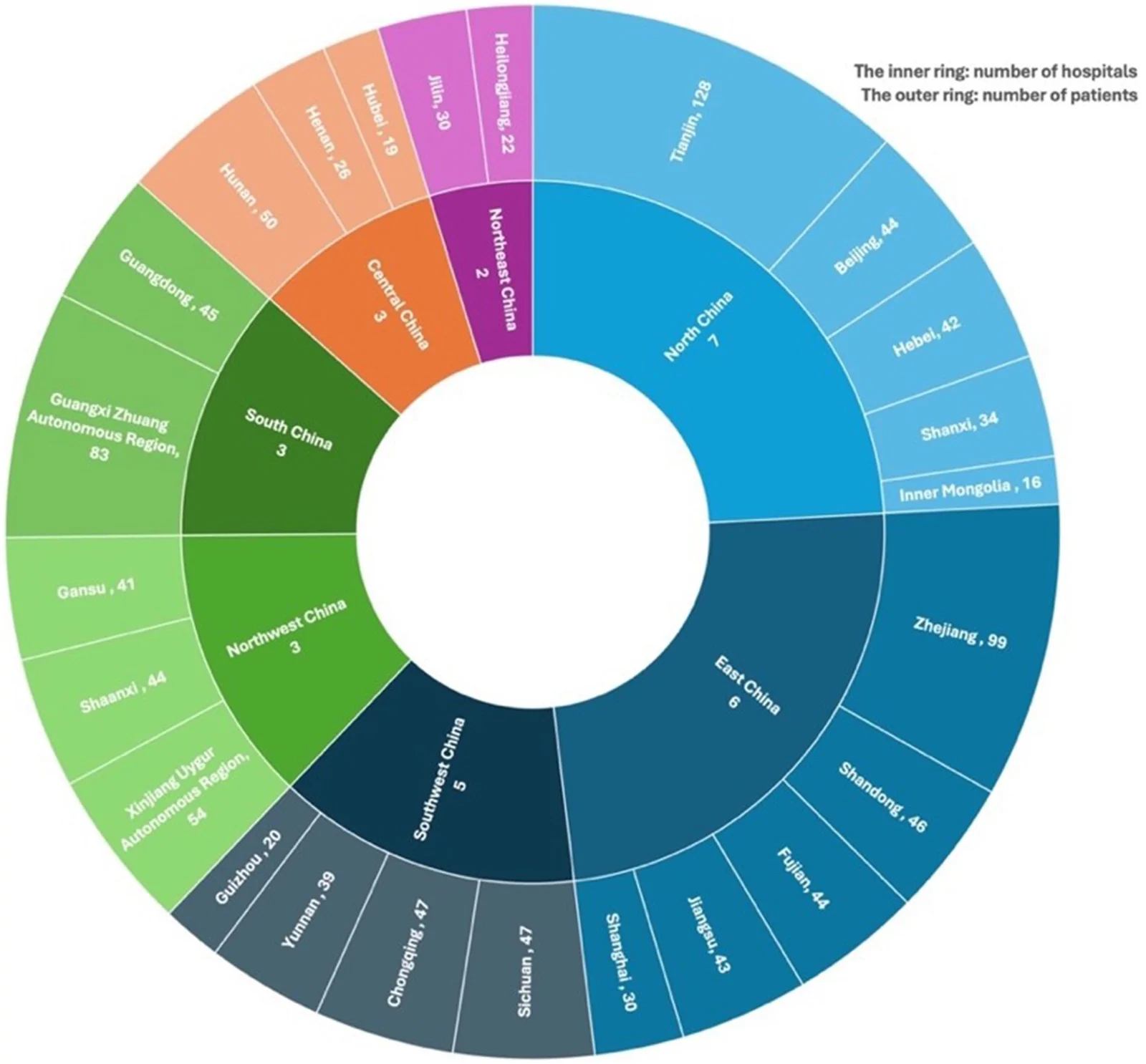
Regional distribution of participating hospitals and enrolled patients.

### Univariate analyses of quality of life according to demographic and clinical characteristics

A total of 1206 questionnaires were collected. After excluding 113 incomplete questionnaires, 1093 participants were included in the final analysis. The mean age of participants was 44.33 years (SD = 12.05), with 41.2% aged ≤40 years, 48.4% between 41 and 60 years, and 10.3% aged ≥61 years. The majority were female (74.7%). Regarding body mass index, 47.3% had normal weight (18.5-23.9 kg/m²), 32.9% were overweight (24.0-27.9 kg/m²), and 15.2% were obese (≥28 kg/m²). Urban residents accounted for 73.5% of the sample. Nearly half of the participants (46.9%) held a college degree or higher, and most were married (84.7%). In terms of employment, 49.3% were workers, followed by retirees (12.9%) and unemployed individuals (11.8%). Most participants were nonsmokers (87.9%) and reported no alcohol consumption (86.6%). Clinically, 78.2% were diagnosed at stage I, and papillary thyroid carcinoma was the predominant pathological type (99.0%). Additional demographic and clinical characteristics are presented in Table 1. Univariate analyses showed that quality of life differed significantly according to gender, body mass index, income level, clinical stage, lymph node metastasis, extent of thyroidectomy, cervical lymph node dissection, sleep disorders, postoperative complications, and regular levothyroxine use (all *P *< .05).

**Table 1.** Univariate analyses of quality of life according to demographic and clinical characteristics (*N *= 1093).

Characteristics		*N*	%	Statistic	*P*
**Age (years.)**	≤40	450	41.2	0.861^a^	.650
	41-60	529	48.4		
	≥61	114	10.3		
**Gender**	Female	817	74.7	−3.089^b^	.002**
	Male	276	25.3		
**BMI (kg/m^2^)**	＜18.5	50	4.6	10.416^a^	.015*
	18.5-23.9	517	47.3		
	24-27.9	360	32.9		
	≥28	166	15.2		
**Residence**	City and Town	803	73.5	−0.014^b^	.989
	Rural	290	26.5		
**Literacy level**	Primary or less	123	11.3	6.241^a^	.100
	Secondary	254	23.2		
	Senior high	203	18.6		
	College degree or above	513	46.9		
**Marital status**	Unmarried	119	10.9	0.099^a^	.992
	Married	926	84.7		
	Divorced	30	2.7		
	Widowed	18	1.6		
**Medical insurance**	Yes	1040	95.2	0.701^a^	.704
	No	41	3.7		
	Others	12	1.1		
**Occupation type**	Student	18	1.6		
	Worker	539	49.3	5.933^a^	.313
	Farmer	118	10.8		
	Jobless	129	11.8		
	Retirement	141	12.9		
	Other	148	13.5		
**Income level (RMB)**	<3000	242	22.1	11.848^a^	.003**
	3001 ∼ 5000	399	36.5		
	>5000	452	41.4		
**Smoking habit**	No	961	87.9	−1.058^b^	.290
	Yes	132	12.1		
**Alcohol consumption**	No	946	86.6	−0.582^b^	.561
	Yes	147	13.4		
**Clinical stage (The eighth edition of the AJCC/TNM staging system)**	I	855	78.2	10.358^a^	.035*
	Ⅱ	99	9.1		
	III	28	2.6		
	IV	26	2.4		
	Unclear	85	7.8		
**Pathological type**	Papillary thyroid carcinoma	1082	99.0	0.849^a^	.838
	Follicular thyroid carcinoma	6	0.5		
	Medullary thyroid carcinoma	3	0.3		
	Anaplastic thyroid carcinoma	2	0.2		
**Lymph node metastasis**	No	710	65.0	−4.936^b^	<.001**
	Yes	383	35.0		
**Extent of thyroidectomy**	Partial	690	63.1	−2.862^b^	.004**
	Total	403	36.9		
**Conventional open surgery**	No	125	11.4	−0.004^b^	.997
	Yes	968	88.6		
**Cervical lymph node dissection**	None	144	13.2	17.970^a^	<.001**
	Central compartment	631	57.7		
	Lateral neck	142	13.0		
	Central compartment and lateral neck	176	16.1		
**Comorbidities**	No	841	76.9	−0.071^b^	.943
	Yes	252	23.1		
**Sleep disorders**	No	731	66.9	−9.190^b^	<.001**
	Yes	362	33.1		
**Complications**	No	740	67.7	−2.058^b^	.040*
	Yes	353	32.3		
**TSH value (mIU/L)**	＜0.1	292	26.7	2.026^a^	.363
	0.1-0.5	113	10.3		
	>1	688	62.9		
**Take levothyroxine regularly**	No	70	6.4	−4.694^b^	<.001**
	Yes	1023	93.6		

Abbreviations: BMI, body mass index; TSH, thyrotropin.

a Kruskal–Wallis *H* test.

b Mann–Whitney *U* test.

* *P *< .05,

** *P *< .01.

### Prevalence and severity of symptoms in patients with thyroid cancer following thyroidectomy

The findings showed that the highest prevalence of fatigue (77.13%), pain (62.76%), disturbed sleep (61.30%), numbness or tingling (56.63%), and hoarseness (55.99%) were observed in patients with thyroid cancer following thyroidectomy. According to the severity of symptoms, the five highest symptoms were in the order of fatigue (2.34 ± 2.16), disturbed sleep (2.00 ± 2.37), hoarseness (1.73 ± 2.28), pain (1.71 ± 1.99), and numbness or tingling (1.62 ± 2.01). The prevalence and severity of symptoms in patients with thyroid cancer are specified in Table 2 (Figure 2).

**Figure 2.**
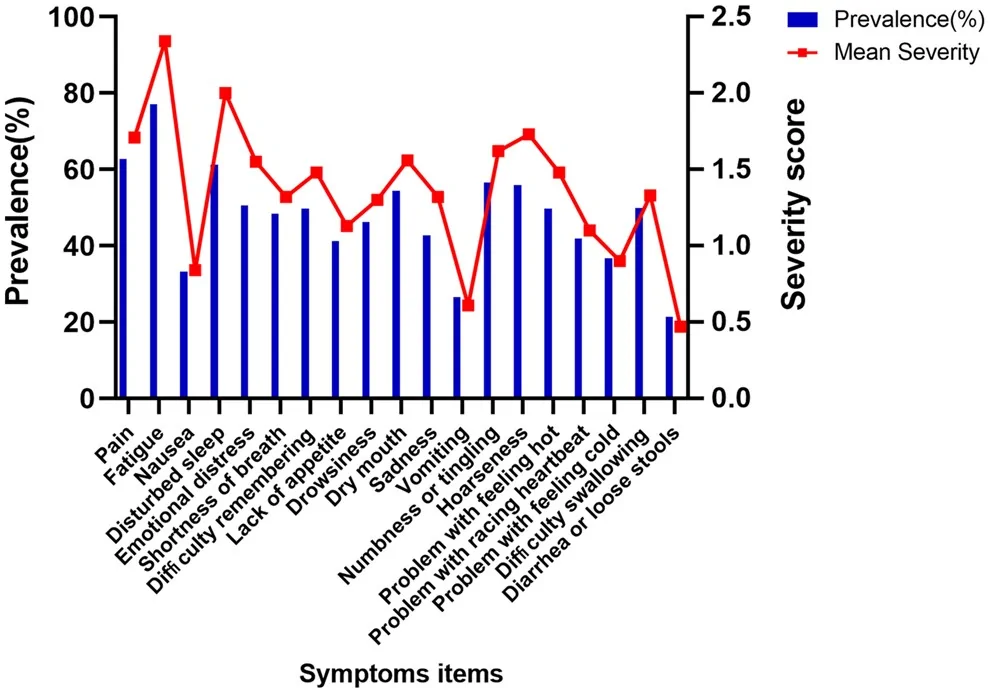
The prevalence and severity of symptoms in patients with thyroid cancer.

**Table 2.** Prevalence and severity of symptoms among patients with thyroid cancer at 1-month postoperative follow-up (*N *= 1093).

Symptoms items	Number of individuals with symptoms (*n*)	Prevalence of symptoms (%)	Symptom severity	
			M (P25, P75)	Mean (SD)
**Pain**	686	62.76	1(0, 3)	1.71 (1.99)
**Fatigue**	843	77.13	2(1, 3)	2.34 (2.16)
**Nausea**	363	33.21	0(0, 1)	0.84 (1.65)
**Disturbed sleep**	670	61.30	1(0, 3)	2.00 (2.37)
**Emotional distress**	552	50.50	1(0, 2)	1.55 (2.16)
**Shortness of breath**	530	48.49	0(0, 2)	1.32 (1.93)
**Difficulty remembering**	544	49.77	0(0, 2)	1.48 (2.08)
**Lack of appetite**	451	41.26	0(0, 2)	1.13 (1.82)
**Drowsiness**	506	46.29	0(0, 2)	1.30 (1.87)
**Dry mouth**	594	54.35	1(0, 2.5)	1.56 (2.07)
**Sadness**	468	42.82	0(0, 2)	1.32 (2.06)
**Vomiting**	290	26.53	0(0, 1)	0.61 (1.39)
**Numbness or tingling**	619	56.63	1(0, 3)	1.62 (2.01)
**Hoarseness**	612	55.99	1(0, 3)	1.73 (2.28)
**Problem with feeling hot**	543	49.68	0(0, 2)	1.48 (2.08)
**Problem with racing heartbeat**	458	41.90	0(0, 2)	1.10 (1.71)
**Problem with feeling cold**	401	36.69	0(0, 1)	0.96 (1.67)
**Difficulty swallowing**	546	49.95	0(0, 2)	1.33 (1.91)
**Diarrhea or loose stools**	234	21.41	0(0, 0)	0.47 (1.17)

### Construction of symptom clusters in patients with thyroid cancer following thyroidectomy

Of the 19 symptoms, diarrhea or loose stools (21.41%) and vomiting (26.53%) had an incidence of less than 30% and were excluded from the symptom cluster analysis. EFA was performed on the remaining 17 symptoms with a KMO value of 0.931 and a Barlett’s spherical test $\chi^{2}$ of 6519.142, *P *< .001, indicating that the data were suitable for factor analysis. The initial EFA results are presented in Table S1, in which several symptoms demonstrated factor loadings < 0.50. Through iterative EFA procedures, symptoms with loadings < 0.50 (dry mouth, problem with feeling hot, and shortness of breath) were sequentially removed. Detailed results of the iterative factor analyses are presented in Tables S2 and S3.

The final EFA identified 3 symptom clusters: psychological-somatic symptom cluster (emotional distress, sadness, nausea, lack of appetite, pain, disturbed sleep, drowsiness, and fatigue), thyroid hormone-related symptom cluster (difficulty remembering, problem with feeling cold, and racing heartbeat), and surgery-related symptom cluster (hoarseness, numbness or tingling, and difficulty swallowing). These 3 symptom clusters explained 60.188% of the total variance. Cronbach’s α coefficients for the psychological-somatic, thyroid hormone-related, and surgery-related symptom clusters were 0.887, 0.718, and 0.624, respectively (Table 3). In addition, the scree plot demonstrated clear inflection points for the first 3 factors with eigenvalues > 1, further supporting retention of a 3-factor solution (Figure S1).

**Table 3.** Exploratory factor analysis of symptom clusters in patients with thyroid cancer at 1-month postoperative follow-up.

Symptom cluster	Symptom	Factor loading		
		psychological-somatic SC	thyroid hormone-related SC	surgery-related SC
	Emotional distress	0.796		
	Sadness	0.763		
	Nausea	0.719		
	Lack of appetite	0.703		
	Pain	0.675		
	Disturbed sleep	0.610		
	Drowsiness	0.596		
	Fatigue	0.587		
	Difﬁculty remembering		0.777	
	Problem with feeling cold		0.774	
	Problem with racing heartbeat		0.542	
	Hoarseness			0.806
	Numbness or tingling			0.611
	Difﬁculty swallowing			0.599
**Cronbach’s α**		0.887	0.718	0.624
**Eigenvalue**		6.315	1.108	1.004
**% of variance**		30.350	16.532	13.305
**% of total variance**		30.350	46.882	60.188

Abbreviation: SC, symptom cluster.

### Multiple regression analysis of quality of life in patients with thyroid cancer following thyroidectomy

Variables that were statistically significant in univariate analyses, together with the scores of the identified symptom clusters, were entered into the multiple linear regression model. Multicollinearity diagnostics indicated no significant collinearity among the independent variables. The final model explained 55.2% of the variance in quality of life (adjusted *R²* = 0.552). The results demonstrated that gender, extent of thyroidectomy, and all 3 symptom clusters were significantly associated with quality of life. Specifically, female patients reported lower quality of life scores than male patients. Patients who underwent total thyroidectomy had lower quality of life scores compared with those who underwent partial thyroidectomy. In addition, greater symptom burden in the psychological-somatic symptom cluster, thyroid hormone-related symptom cluster, and surgery-related symptom cluster was significantly associated with poorer quality of life. Among these, the psychological-somatic symptom cluster showed the strongest association with quality of life (β = −0.596, *P *< .001). Detailed results are presented in Table 4.

**Table 4.** Multiple linear regression analysis of factors associated with quality of life (*N *= 1093).

Variables	*B* (95% CI)	*SE*	β	*t*	*P*
**Constant**	94.872	1.943		48.827	<.001
**Psychological-somatic SC**	−0.647 (−0.713, −0.581)	0.033	−0.596	−19.322	<.001**
**Thyroid hormone-related SC**	−0.393 (−0.556, −0.229)	0.083	−0.131	−4.718	<.001**
**Surgery-related SC**	−0.183 (−0.335, −0.030)	0.078	−0.065	−2.354	.019*
**Gender (male as reference)**					
** Female**	−1.524 (−2.795, −0.253)	−0.648	−0.051	−2.353	.019*
**Lymph node metastasis (none as reference)**					
** Yes**	−0.693 (−1.916, 0.529)	0.623	−0.025	−1.112	.266
**Type of thyroidectomy (partial as reference)**					
** Total**	−1.568 (−2.746, −0.390)	0.600	−0.058	−2.611	.009**
**Sleep disorders (none as reference)**					
** Yes**	0.063 (−1.137, 1.263)	0.612	0.002	0.103	.918
**Complication (none as reference)**					
** Yes**	0.370 (−0.885, 1.626)	0.640	0.013	0.579	.563
**Take levothyroxine regularly (irregular as reference)**					
** Regular**	1.848 (−0.348, 4.045)	1.119	0.035	1.651	.099
**BMI (<18.5 as reference)**					
** 18.5-23.9**	0.658 (−1.928, 3.245)	1.318	0.025	0.499	.618
** 24-27.9**	0.272 (−2.369, 2.912)	1.346	0.010	0.202	.840
** ≥28**	1.258 (−1.577, 4.094)	1.445	0.034	0.871	.384
**Income level (<3000 as reference)**					
** 3001-5000**	−0.502 (−1.931, 0.927)	0.728	−0.018	−0.690	.491
** >5000**	0.593 (−0.815, 2.002)	0.718	0.022	0.827	.409
**Lateral neck dissection (none as reference)**					
** Central compartment**	−0.684 (−2.348, 0.980)	0.848	−0.026	−0.806	.420
** Lateral neck**	−1.303 (−3.433, 0.828)	1.086	−0.033	−1.200	.231
** Central compartment and lateral neck**	−1.692 (−3.871, 0.488)	1.111	−0.047	−1.523	.128
**Cancer stage (stage I as reference)**					
** Stage Ⅱ**	0.230 (−1.645, 2.104)	0.955	0.005	0.240	.810
** Stage III**	0.191 (−3.203, 3.586)	1.730	0.002	0.111	.912
** Stage IV**	0.816 (−3.714, 4.345)	1.799	0.009	0.453	.650
** Unclear**	1.327 (−0.669, 3.322)	1.017	0.027	1.305	.192

Abbreviation: SC, symptom cluster.

*F *= 65.064, *P *< .001, *R*^2^ = 0.561, adjusted *R*^2^ = 0.552.

* *P *< .05,

** *P *< .01.

## Discussion

In this study, fatigue and disturbed sleep were the symptoms with the highest prevalence and severity among patients following thyroidectomy, which is consistent with findings from previous studies.^9^^,^^25–27^ These symptoms may reflect complex physiological and neuroendocrine responses during the early postoperative recovery period. Surgical trauma and postoperative tissue repair can induce substantial physiological stress and increased energy expenditure.^28^^,^^29^ In addition, fluctuations in thyroid hormone levels following partial or total thyroidectomy may disrupt metabolic homeostasis and neuroendocrine regulation.^26^^,^^30^ Such hormonal instability may further affect autonomic nervous system activity, circadian regulation, and neural excitability, thereby contributing to fatigue, disturbed sleep, and related somatic symptoms. These endocrine and physiological fluctuations are commonly observed during the first postoperative month, a period characterized by ongoing functional adaptation and recovery.^31^

Beyond the high prevalence of individual symptoms, our findings further demonstrated that symptoms during early survivorship tended to co-occur as distinct symptom clusters, potentially reflecting shared biological, neuroendocrine, and psychophysiological processes. The psychological-somatic symptom cluster may be associated with postoperative stress responses, endocrine fluctuations, and disrupted autonomic regulation,^32^^,^^33^ which together contribute to the coexistence of emotional distress, fatigue, sleep disturbance, pain, and appetite-related symptoms. Persistent physical symptoms may aggravate emotional distress, whereas psychological stress may further amplify symptom perception and physiological dysregulation, resulting in dynamic interactions among symptoms and a cycle of mutual reinforcement. The thyroid hormone-related symptom cluster, including racing heartbeat, difficulty remembering, and feeling cold, may reflect altered thyroid hormone feedback regulation and impaired neuroendocrine adaptation following thyroidectomy. Thyroid hormones play important roles in autonomic regulation,^32^ cognitive function,^34^ and energy metabolism.^35^ Postoperative fluctuations in thyroid hormone levels may disrupt neurotransmitter activity, neuronal energy metabolism, and hypothalamic–pituitary–thyroid axis feedback mechanisms, thereby affecting both neural and cognitive functioning. These interconnected neuroendocrine processes may contribute to the simultaneous occurrence of cardiovascular, cognitive, and thermoregulatory symptoms observed in this cluster.

In contrast, the surgery-related symptom cluster, comprising hoarseness, numbness or tingling, and difficulty swallowing, appears to be more directly associated with surgical trauma, transient neural irritation, and postoperative functional adaptation.^36^^,^^37^ These shared anatomical vulnerabilities may explain why these symptoms tend to cluster together and support the notion that surgery-related symptoms represent a distinct mechanistic symptom cluster following thyroidectomy. Variations in symptom cluster composition across studies were observed and may be attributable to differences in study populations, symptom assessment tools, and survivorship stages. For example, Cai et al.^38^ primarily included patients with advanced thyroid cancer, whereas our study focused on patients during the early postoperative recovery period. Li et al.^14^ assessed symptom experiences using the EORTC QLQ-THY34 during the immediate postoperative period (1-3 days after surgery), whereas our study used the MDASI-THY and evaluated patients at one month after thyroidectomy. Despite these methodological and temporal differences, symptoms related to voice changes, swallowing dysfunction, and sensory disturbances were repeatedly grouped within the same symptom cluster.^14^ Such consistency suggests that surgery-related symptoms may reflect a relatively robust and clinically relevant symptom cluster during early survivorship in patients with thyroid cancer. Although the internal consistency of this cluster was modest (Cronbach’s α = 0.624), this may partly reflect the fact that the cluster comprised only 3 symptoms, together with the heterogeneous nature of these postoperative manifestations. Hoarseness, numbness or tingling, and difficulty swallowing represent distinct functional impairments but are among the most common complications following thyroidectomy. Despite this, considering these symptoms together may still be clinically meaningful for postoperative symptom assessment and management.

The present study further demonstrated that all 3 symptom clusters were significantly associated with poorer quality of life, highlighting the multidimensional burden of co-occurring symptoms during early survivorship. This finding is consistent with previous studies in patients with lung,^39^ gastric,^40^ esophageal,^41^ and breast cancer,^42^ in which symptom clusters were consistently associated with poorer quality of life compared with isolated symptoms. Symptoms within a cluster may interact dynamically and reinforce one another through shared biological and psychological pathways, thereby amplifying their overall burden. Among the identified clusters, the psychological-somatic symptom cluster exhibited the strongest association with quality of life, which is consistent with the findings reported by Luo et al.^43^ One possible explanation is that this cluster encompasses symptoms spanning multiple domains, including emotional distress, pain, fatigue, sleep disturbance, and appetite-related symptoms. The coexistence of these symptoms across multiple domains may help explain why the psychological-somatic symptom cluster showed the strongest association with poorer quality of life in the present study. Furthermore, psychological distress and physical symptoms are closely interconnected. Psychological stress may influence somatic functioning through neuroendocrine and autonomic pathways, whereas persistent physical symptoms may further exacerbate emotional distress, creating a cycle of mutual reinforcement. For example, pain may increase negative emotions and heighten symptom perception,^44^^,^^45^ while disturbed sleep and fatigue frequently interact bidirectionally, with poor sleep contributing to fatigue and fatigue further impairing sleep quality.^9^ Such symptom interactions may increase a greater symptom burden, which is associated with poorer quality of life. The thyroid hormone-related symptom cluster was also associated with poorer quality of life, although its impact was less pronounced than that of the psychological-somatic symptom cluster. Previous studies have shown that thyroid dysfunction and TSH suppression therapy may be associated with cognitive complaints, cardiovascular symptoms, and impaired well-being among patients with thyroid cancer.^46^^,^^47^ These symptoms may limit physical activity,^48^ interfere with work and social participation, and increase emotional concerns regarding health status.^49^ The surgery-related symptom cluster was likewise associated with poorer quality of life. This cluster included hoarseness, numbness or tingling, and difficulty swallowing, all of which are common postoperative complications that directly affect patients’ ability to communicate, eat, and perform routine daily activities. These symptoms may also cause frustration, social embarrassment, and concerns regarding recovery, thereby extending their association beyond physical functioning alone.^50–52^ Previous studies have similarly reported that persistent voice, swallowing, and sensory disturbances following thyroidectomy are associated with reduced quality of life and delayed functional recovery.^53^^,^^54^ Therefore, early identification and management of surgery-related symptoms remain important components of postoperative survivorship care.

In addition to symptom burden, female sex and total thyroidectomy were identified as factors associated with poorer quality of life in the present study, consistent with previous studies in patients with thyroid cancer.^27^^,^^55^ Female patients reported lower quality of life scores. One possible explanation is that women tend to experience greater psychological distress and symptom burden following cancer diagnosis and treatment.^56^ Furthermore, because thyroid cancer predominantly affects women of reproductive age, concerns regarding fertility,^57^ family responsibilities,^58^ and disease recurrence^59^ may impose additional burdens during recovery. Patients who underwent total thyroidectomy also reported poorer quality of life. Compared with partial thyroidectomy, total thyroidectomy is associated with lifelong dependence on thyroid hormone replacement therapy^60^ and a higher risk of postoperative complications, including hypocalcemia and voice-related problems.^61^ Collectively, these factors may complicate postoperative recovery and adversely affect quality of life.

### Strengths and limitations

This study offers numerous notable strengths. First, it included a large sample from 29 hospitals across all seven geographic regions of China, providing strong nationwide representativeness and enhancing generalizability. Second, it specifically focused on the early survivorship following thyroidectomy, a critical yet underexplored period in symptom cluster research. Third, the use of a validated symptom assessment tool enabled robust identification of symptom clusters.

However, several limitations should be considered when interpreting the findings. First, the cross‑sectional design cannot establish the temporal sequence and causal relationships between symptom clusters and quality of life. Longitudinal studies are necessary to investigate changes in symptom clusters patterns and their long-term effects on patient well-being. Second, the use of convenience sampling may have introduced selection bias, and the reliance on self-administered questionnaires could be subject to participants’ subjective interpretation. Third, some symptom domains assessed by the MDASI-THY, including fatigue, pain, and disturbed sleep, overlap conceptually with those included in the EORTC QLQ-C30. This overlap may have introduced shared variance and partially inflated the observed associations between symptom clusters and quality of life. Fourth, the identified symptom cluster structure was not internally validated (eg, using split-sample confirmatory factor analysis) or externally validated in an independent population. Therefore, the stability and reproducibility of the identified symptom cluster structure require further confirmation in future studies. Fifth, all participants were recruited from China. Although the multicenter design enhanced the representativeness of the sample within the Chinese population, cultural, social, and healthcare-system differences may influence symptom perception, reporting behaviors, and quality of life outcomes. Therefore, caution should be exercised when generalizing these findings to populations from other cultural backgrounds.

## Conclusions

This national multicenter cross-sectional study identified 3 distinct symptom clusters among patients with thyroid cancer during early survivorship: a psychological-somatic symptom cluster, a thyroid hormone-related symptom cluster, and a surgery-related symptom cluster. All 3 symptom clusters were significantly associated with poorer quality of life, with the psychological-somatic symptom cluster showing the strongest association. In addition, female sex and total thyroidectomy were identified as factors associated with poorer quality of life. These findings provide further evidence that symptom experiences during early survivorship are multidimensional and tend to occur in clusters rather than as isolated symptoms. Early identification of symptom clusters and high-risk patient groups may facilitate more targeted postoperative assessment and management. Future longitudinal studies are warranted to validate the stability of symptom clusters over time and clarify their long-term associations with quality of life. Building on the present findings, multicenter interventional studies are needed to determine whether symptom cluster–guided management can effectively reduce symptom burden and improve quality of life during early survivorship after thyroidectomy.

## Supplementary Material

oyag313_Supplementary_Data

## Data Availability

The dataset of this study is available from the corresponding author on reasonable request.

## References

[1] Lortet-Tieulent J , Franceschi S, Dal Maso L, Vaccarella S. Thyroid cancer “epidemic” also occurs in low- and Middle-income countries. Int J Cancer. 2019;144:2082-2087. 10.1002/ijc.31884 PMC6587710 30242835

[2] Bray F , Laversanne M, Sung H, et al Global cancer statistics 2022: GLOBOCAN estimates of incidence and mortality worldwide for 36 cancers in 185 countries. CA Cancer J Clin. 2024;74:229-263. 10.3322/caac.21834 38572751

[3] Han B , Zheng R, Zeng H, et al Cancer incidence and mortality in China, 2022. J Natl Cancer Cent. 2024;4:47-53. 10.1016/j.jncc.2024.01.006 PMC11256708 39036382

[4] Zeng H , Zheng R, Sun K, et al Cancer survival statistics in China 2019-2021: a multicenter, population-based study. J Natl Cancer Cent. 2024;4:203-213. 10.1016/j.jncc.2024.06.005 PMC11401485 39281724

[5] Van Spall HGC , Rahman T, Mytton O, et al Comparative effectiveness of transitional care services in patients discharged from the hospital with heart failure: a systematic review and network meta-analysis. Eur J Heart Fail. 2017;19:1427-1443. 10.1002/ejhf.765 28233442

[6] Naylor MD , Marcille J. Managing the transition from the hospital. Manag Care. 2014;23:27-30. 25109044

[7] Kaltsas G , Vgontzas A, Chrousos G. Fatigue, endocrinopathies, and metabolic disorders. PM R. 2010;2:393-398. 10.1016/j.pmrj.2010.04.011 20656620

[8] To J , Goldberg AS, Jones J, et al A systematic review of randomized controlled trials for management of persistent post-treatment fatigue in thyroid cancer survivors. Thyroid. 2015;25:198-210. 10.1089/thy.2014.0418 25382050

[9] Chan W-L , Choi HC-W, Lang B, et al Health-related quality of life in Asian differentiated thyroid cancer survivors. Cancer Control. 2021;28:10732748211029726. 10.1177/10732748211029726 PMC8252343 34189945

[10] Goswami S , Mongelli M, Peipert BJ, et al Benchmarking health-related quality of life in thyroid cancer versus other cancers and United States normative data. Surgery. 2018;164:986-992. 10.1016/j.surg.2018.06.042 30149935

[11] Singer S , Husson O, Tomaszewska IM, et al Quality-of-life priorities in patients with thyroid cancer: a multinational European Organisation for Research and Treatment of Cancer phase I study. Thyroid. 2016;26:1605-1613. 10.1089/thy.2015.0640 27605136

[12] Chen C , Cao J, Wang Y, et al Health-related quality of life and thyroid cancer-specific symptoms in patients treated for differentiated thyroid cancer: a single-center cross-sectional survey from mainland China. Thyroid. 2023;33:474-483. 10.1089/thy.2022.0490 36792949

[13] Miaskowski C , Barsevick A, Berger A, et al Advancing symptom science through symptom cluster research: expert panel proceedings and recommendations. J Natl Cancer Inst. 2017;109:djw253. 10.1093/jnci/djw253 PMC5939621 28119347

[14] Li Y , Sun L, Wu M, et al Correlation between early postoperative symptom groups and quality of life in patients with thyroid cancer. Chin Arch Otolaryngol Head Neck Surg. 2024;31:485-491. 10.16066/j.1672-7002.2024.08.002

[15] de Rooij BH , Oerlemans S, van Deun K, et al Symptom clusters in 1330 survivors of 7 cancer types from the PROFILES registry: a network analysis. Cancer. 2021;127:4665-4674. 10.1002/cncr.33852 PMC9291877 34387856

[16] Cho S , Tutelman P, Chomistek T, et al “No such thing as good cancer”: a qualitative exploration of the experience of early-onset thyroid cancer in survivors. Support Care Cancer. 2024;32:671. 10.1007/s00520-024-08850-3 39292348

[17] Randle RW , Bushman NM, Orne J, et al Papillary thyroid cancer: the good and bad of the “good cancer.” Thyroid. 2017;27:902-907. 10.1089/thy.2016.0632 PMC5561445 28510505

[18] National health commission of the People’s Republic of China Medical Administration and Hospital Administration (2022) guidelines for the diagnosis and treatment of thyroid carcinoma. Chin J Pract Surg. 1363;42:1343-1357. 10.19538/j.cjps.issn1005-2208.2022.12.02

[19] Gning I , Trask PC, Mendoza TR, et al Development and initial validation of the thyroid cancer module of the M. D. Anderson Symptom Inventory. Oncology. 2009;76:59-68. 10.1159/000178809 19052478

[20] Hu Z-Y , Gou J-X, Cai M, Zhang Y-E. Translation and validation of M.D. Anderson Symptom Inventory-Thyroid cancer module in Chinese thyroid cancer patients: a cross-sectional and methodological study. BMC Cancer. 2022;22:924. 10.1186/s12885-022-09995-2 PMC9419372 36028793

[21] Aaronson NK , Ahmedzai S, Bergman B, et al The European Organization for Research and Treatment of Cancer QLQ-C30: a quality-of-life instrument for use in international clinical trials in oncology. J Natl Cancer Inst. 1993;85:365-376. 10.1093/jnci/85.5.365 8433390

[22] Wan C , Chen M, Zhang C, et al Review of the Chinese version of the quality of life scale for cancer patients EORTC QLQ-C30. J Practical Oncol. 2005;353-355.

[23] Giesinger JM , Kieffer JM, Fayers PM, et al EORTC Quality of Life Group Replication and validation of higher order models demonstrated that a summary score for the EORTC QLQ-C30 is robust. J Clin Epidemiol. 2016;69:79-88. 10.1016/j.jclinepi.2015.08.007 26327487

[24] Qin Y , Chen J, Li Y, et al Bridging symptoms linking postoperative symptom clusters seven days after esophagectomy: a multicenter network analysis of 418 patients with esophageal cancer. Asia Pac J Oncol Nurs. 2026;13:100830. 10.1016/j.apjon.2025.100830 PMC12796605 41536886

[25] Trivedi R , Man H, Madut A, et al Irregular sleep/wake patterns are associated with reduced quality of life in post-treatment cancer patients: a study across three cancer cohorts. Front Neurosci. 2021;15:700923. 10.3389/fnins.2021.700923 PMC8494030 34630009

[26] Maki Y , Horiuchi K, Okamoto T. Fatigue and quality of life among thyroid cancer survivors without persistent or recurrent disease. Endocr Connect. 2022;11:e210506. 10.1530/EC-21-0506 PMC8942325 35107083

[27] Chen W , Li J, Peng S, et al Association of total thyroidectomy or thyroid lobectomy with the quality of life in patients with differentiated thyroid cancer with low to intermediate risk of recurrence. JAMA Surg. 2022;157:200-209. 10.1001/jamasurg.2021.6442 PMC8696698 34935859

[28] Bower JE. Cancer-related fatigue—mechanisms, risk factors, and treatments. Nat Rev Clin Oncol. 2014;11:597-609. 10.1038/nrclinonc.2014.127 PMC4664449 25113839

[29] Perrier J , Giffard B, Wu LM, et al Editorial: Sleep and circadian rhythms in cancer patients and their relationship with quality of life. Front Neurosci. 2022;16:1060184. 10.3389/fnins.2022.1060184 PMC9742586 36518532

[30] Zhou L , Li S, Wu Y, et al Significant dysregulation of lipid metabolism in patients with papillary thyroid carcinoma after thyroidectomy. Front Endocrinol (Lausanne). 2023;14:1223527. 10.3389/fendo.2023.1223527 PMC10602777 37900134

[31] Dou Y , Chen Y, Hu D, Su X. The recovery of thyroid function in low-risk papillary thyroid cancer after lobectomy: a 3-year follow-up study. Front Endocrinol (Lausanne). 2020;11:619841. 10.3389/fendo.2020.619841 PMC7899978 33633689

[32] Haymart P , Levin NJ, Haymart MR. The psychosocial impact of thyroid cancer. Curr Opin Endocrinol Diabetes Obes. 2023;30:252-258. 10.1097/MED.0000000000000815 PMC10526714 37288721

[33] Luo S , Wei X, Zhao J, et al Effect of psychosomatic symptom intervention on psychosomatic symptoms during initial treatment in patients with differentiated thyroid cancer: a single-blind randomized controlled trial. Cancer Nurs. 2025. 10.1097/NCC.0000000000001511 40266664

[34] Yaniv D , Vainer I, Amir I, et al Quality of life following lobectomy versus total thyroidectomy is significantly related to hypothyroidism. J Surg Oncol. 2022;126:640-648. 10.1002/jso.26983 PMC9544480 35689620

[35] López M , Varela L, Vázquez MJ, et al Hypothalamic AMPK and fatty acid metabolism mediate thyroid regulation of energy balance. Nat Med. 2010;16:1001-1008. 10.1038/nm.2207 PMC2935934 20802499

[36] Gür EO , Haciyanli M, Karaisli S, et al Intraoperative nerve monitoring during thyroidectomy: evaluation of signal loss, prognostic value and surgical strategy. Ann R Coll Surg Engl. 2019;101:589-595. 10.1308/rcsann.2019.0087 PMC6818062 31219340

[37] Su D , Zhang Z, Xia F, Li X. The safety, benefits and future development of overnight and outpatient thyroidectomy. Front Endocrinol (Lausanne). 2023;14:1110038. 10.3389/fendo.2023.1110038 PMC10113618 37091845

[38] Cai M , Gou J. Symptom clusters of patients with advanced thyroid cancer: a cross-sectional study. Endocrine. 2025;87:718-723. 10.1007/s12020-024-04051-4 39320592

[39] Yang X , Bai J, Zhang J, et al Symptom clusters and their impacts on the quality of life of patients with lung cancer receiving immunotherapy: a cross-sectional study. J Clin Nurs. 2025;34:1725-1740. 10.1111/jocn.17321 38886988

[40] Fu L , Feng X, Jin Y, et al Symptom clusters and quality of life in gastric cancer patients receiving chemotherapy. J Pain Symptom Manage. 2022;63:230-243. 10.1016/j.jpainsymman.2021.09.003 34537311

[41] Wang Y , Xie Z, Liu Y, et al Symptom clusters and impact on quality of life in esophageal cancer patients. Health Qual Life Outcomes. 2022;20:168. 10.1186/s12955-022-02084-9 PMC9783739 36564827

[42] Jing F , Zhu Z, Qiu J, et al Symptom clusters and quality of life in patients with breast cancer receiving endocrine therapy in China. Semin Oncol Nurs. 2023;39:151443. 10.1016/j.soncn.2023.151443 37173235

[43] Luo Y , Zhang L, Mao D, et al Symptom clusters and impact on quality of life in lung cancer patients undergoing chemotherapy. Qual Life Res. 2024;33:3363-3375. 10.1007/s11136-024-03778-x PMC11599356 39240422

[44] Su N , Lobbezoo F, van Wijk A, et al Associations of pain intensity and pain-related disability with psychological and socio-demographic factors in patients with temporomandibular disorders: a cross-sectional study at a specialised dental clinic. J Oral Rehabil. 2017;44:187-196. 10.1111/joor.12479 28036120

[45] Murray I , Bhanot G, Bhargava A. Neuron-glia-immune triad and cortico-limbic system in pathology of pain. Cells. 2021;10:1553. 10.3390/cells10061553 PMC8234386 34205372

[46] He J , Li J, Cheng Y, et al Guidelines of Chinese Society of Clinical Oncology(CSCO) differentiated thyroid cancer. J Cancer Control Treatment. 2021;34:1164-1201.

[47] Jin S , Yang Y-T, Bao W, et al Naming difficulties after thyroid stimulating hormone suppression therapy in patients with differentiated thyroid carcinoma: a prospective cohort study. Endocrine. 2019;65:327-337. 10.1007/s12020-019-01943-8 PMC6656796 31056722

[48] Basiura K , Starzomska D, Rymaszewska K, et al The association between hypothyroidism, mental disorders and physical activity: a comprehensive review. Qual Sport. 2024;16:52499-52499. 10.12775/QS.2024.16.52499

[49] Jurado-Flores M , Warda F, Mooradian A. Pathophysiology and clinical features of neuropsychiatric manifestations of thyroid disease. J Endocr Soc. 2022;6:bvab194. 10.1210/jendso/bvab194 PMC8765786 35059548

[50] Park JS , Frank E, Simental A, et al Incidence of dysphonia and dysphagia exceeds recurrent laryngeal nerve injury during thyroid surgery. J Voice. 2023;37:616-620. 10.1016/j.jvoice.2021.02.030 34053823

[51] Mihai R. Voice and swallowing symptoms after thyroid surgery assessed using the ThyVoice online platform. Eur Thyroid J. 2023;12:e230008. 10.1530/ETJ-23-0008 PMC10305707 37115021

[52] Scerrino G , Tudisca C, Bonventre S, et al (2017) Swallowing disorders after thyroidectomy: What we know and where we are. A systematic review. Int J Surg 41 Suppl 1: S94-S102. 10.1016/j.ijsu.2017.03.078 28506421

[53] Kim G-J , Bang J, Shin H-I, et al Persistent subjective voice symptoms for two years after thyroidectomy. Am J Otolaryngol. 2023;44:103820. 10.1016/j.amjoto.2023.103820 36893530

[54] Lombardi CP , Raffaelli M, De Crea C, et al Long-term outcome of functional post-thyroidectomy voice and swallowing symptoms. Surgery. 2009;146:1174-1181. 10.1016/j.surg.2009.09.010 19958946

[55] Goldfarb M , Casillas J. Thyroid cancer-specific quality of life and health-related quality of life in young adult thyroid cancer survivors. Thyroid. 2016;26:923-932. 10.1089/thy.2015.0589 27161396

[56] Gao J , Wang X, Zhang L, et al Prevalence and predictors of psychological distress among patients with thyroid cancer during transitional period in China: a cross-sectional study. Support Care Cancer. 2022;30:7903-7911. 10.1007/s00520-022-07225-w 35727374

[57] Lai F , Chen W, Zhang J, et al Reproductive concern, fertility intention, and pregnancy outcomes in young women with thyroid cancer after thyroid surgery: a prospective cohort study. Thyroid. 2025;35:691-700. 10.1089/thy.2024.0660 40402840

[58] Kuswanto CN , Stafford L, Sharp J, Schofield P. Psychological distress, role, and identity changes in mothers following a diagnosis of cancer: a systematic review. Psychooncology. 2018;27:2700-2708. 10.1002/pon.4904 30289196

[59] Bresner L , Banach R, Rodin G, et al Cancer-related worry in Canadian thyroid cancer survivors. J Clin Endocrinol Metab. 2015;100:977-985. 10.1210/jc.2014-3169 PMC4333046 25393643

[60] Chiardi I , Croce L, Caccavale P, et al Establishing the adequate levothyroxine dose after total thyroidectomy: a systematic review with meta-analysis. J Clin Endocrinol Metab. 2025;110:3301-3308. 10.1210/clinem/dgaf417 PMC12527440 40682434

[61] Jeon YW , Gwak HG, Lim ST, et al Long-term prognosis of unilateral and multifocal papillary thyroid microcarcinoma after unilateral lobectomy versus total thyroidectomy. Ann Surg Oncol. 2019;26:2952-2958. 10.1245/s10434-019-07482-w 31264119

