# Supplementary material for: Symptom clusters and quality of life among patients with thyroid cancer during early survivorship: a national multicenter cross-sectional study
Source: Oncologist. 2026 Sep 24;31(10):oyag313. doi: 10.1093/oncolo/oyag313 (PMC13628382; doi:10.1093/oncolo/oyag313)
Supplement: Supplementary file 1 [file oyag313_supplementary_data.zip › Supplementary table.docx]

Supplementary table S1	The results of the first exploratory factor analysis

| Symptom | Factor loading | | |
| --- | --- | --- | --- |
|  | Factor 1 | Factor 2 | Factor 3 |
| Emotional distress | 0.783 |  |  |
| Sadness | 0.753 |  |  |
| Nausea | 0.713 |  |  |
| Lack of appetite | 0.710 |  |  |
| Pain | 0.673 |  |  |
| Disturbed sleep | 0.603 |  |  |
| Drowsiness | 0.588 |  |  |
| Fatigue | 0.584 |  |  |
| Shortness of breath | 0.472 ^a^ |  |  |
| Dry mouth | 0.448^a^ |  |  |
| Difﬁculty remembering |  | 0.773 |  |
| Problem with feeling cold |  | 0.753 |  |
| Problem with racing heartbeat |  | 0.556 |  |
| Problem with feeling hot |  | 0.472 ^a^ |  |
| Hoarseness |  |  | 0.809 |
| Difﬁculty swallowing |  |  | 0.612 |
| Numbness or tingling |  |  | 0.559 |
| Cronbach’s α | 0.895 | 0.757 | 0.624 |
| Eigenvalue | 7.592 | 1.178 | 1.004 |
| % of variance | 27.218 | 16.606 | 13.674 |
| % of total variance | 27.218 | 43.824 | 57.497 |

“^a^”: symptom loading＜0.5

Supplementary table S2	The results of the second exploratory factor analysis

| Symptom | Factor loading | | |
| --- | --- | --- | --- |
|  | Factor 1 | Factor 2 | Factor 3 |
| Emotional distress | 0.785 |  |  |
| Sadness | 0.755 |  |  |
| Nausea | 0.713 |  |  |
| Lack of appetite | 0.712 |  |  |
| Pain | 0.672 |  |  |
| Disturbed sleep | 0.604 |  |  |
| Drowsiness | 0.591 |  |  |
| Fatigue | 0.585 |  |  |
| Shortness of breath | 0.474^a^ |  |  |
| Difﬁculty remembering |  | 0.776 |  |
| Problem with feeling cold |  | 0.760 |  |
| Problem with racing heartbeat |  | 0.555 |  |
| Problem with feeling hot |  | 0.464^a^ |  |
| Hoarseness |  |  | 0.811 |
| Difﬁculty swallowing |  |  | 0.613 |
| Numbness or tingling |  |  | 0.560 |
| Cronbach’s $\boldsymbol{\alpha}$ | 0.895 | 0.757 | 0.624 |
| Eigenvalue | 7.162 | 1.175 | 1.004 |
| % of variance | 27.821 | 16.664 | 13.898 |
| % of total variance | 27.821 | 44.485 | 58.383 |

“^a^”: symptom loading＜0.5

Supplementary table S3	The results of the third exploratory factor analysis

| Symptom | Factor loading | | |
| --- | --- | --- | --- |
|  | Factor 1 | Factor 2 | Factor 3 |
| Emotional distress | 0.790 |  |  |
| Sadness | 0.759 |  |  |
| Nausea | 0.719 |  |  |
| Lack of appetite | 0.695 |  |  |
| Pain | 0.682 |  |  |
| Disturbed sleep | 0.607 |  |  |
| Drowsiness | 0.592 |  |  |
| Fatigue | 0.580 |  |  |
| Shortness of breath | 0.455^a^ |  |  |
| Difﬁculty remembering |  | 0.790 |  |
| Problem with feeling cold |  | 0.760 |  |
| Problem with racing heartbeat |  | 0.547 |  |
| Hoarseness |  |  | 0.825 |
| Difﬁculty swallowing |  |  | 0.602 |
| Numbness or tingling |  |  | 0.579 |
| Cronbach’s $\boldsymbol{\alpha}$ | 0.895 | 0.718 | 0.624 |
| Eigenvalue | 6.814 | 1.122 | 1.004 |
| % of variance | 29.319 | 16.835 | 13.447 |
| % of total variance | 29.319 | 46.154 | 59.601 |

“^a^”: symptom loading＜0.5
