# Supplementary material for: Symptom clusters and quality of life among patients with thyroid cancer during early survivorship: a national multicenter cross-sectional study
Source: Oncologist. 2026 Sep 24;31(10):oyag313. doi: 10.1093/oncolo/oyag313 (PMC13628382; doi:10.1093/oncolo/oyag313)
Supplement: Supplementary file 1 [file oyag313_supplementary_data.zip › Supplementary figure.docx]

Supplementary figure 1: Scatter plot of symptom clusters in patients after thyroidectomy
